# Ethyl 4-hydr­oxy-9-tosyl-9*H*-carbazole-3-carboxyl­ate

**DOI:** 10.1107/S1600536809021035

**Published:** 2009-06-06

**Authors:** Tuncer Hökelek, Hakan Dal, Barış Tercan, Sibel Gülle, Yavuz Ergün

**Affiliations:** a Department of Physics, Hacettepe University, 06800 Beytepe, Ankara, Turkey; b Department of Chemistry, Faculty of Science, Anadolu University, 26470 Yenibağlar, Eskişehir, Turkey; c Department of Physics, Karabük University, 78050, Karabük, Turkey; d Department of Chemistry, Faculty of Arts and Sciences, Dokuz Eylül University, Tınaztepe, 35160 Buca-Izmir, Turkey

## Abstract

In the title compound, C_22_H_19_NO_5_S, the carbazole skeleton is nearly planar [maximum deviation = 0.043 (1) Å] with the pyrrole ring oriented at dihedral angles of 2.32 (6) and 1.77 (6)° with respect to the adjacent benzene rings. The dihedral angle between the benzene ring of the tosyl group and the carbazole skeleton is 82.25 (5)°. Intra­molecular O—H⋯O hydrogen bonding results in the formation of a planar six-membered ring, which is oriented at a dihedral angle of 3.06 (4)° with respect to the adjacent carbazole skeleton. In the crystal structure, weak inter­molecular C—H⋯O inter­actions link the mol­ecules into infinite chains and π–π contacts between the benzene rings and between the pyrrole and benzene rings [centroid–centroid distances = 3.374 (1) and 3.730 (1) Å, respectively] may further stabilize the structure. A weak C—H⋯π inter­action is also present.

## Related literature

For the use of tetra­hydro­carbazolone derivatives in the synthesis of Ondansetron, an anti­emetic drug inhibiting the serotonin 5-HT_3_ receptor, see: Coates *et al.* (1987 ▶); Gutman & Cyjon (2006 ▶); Molnar *et al.* (2006 ▶). Tetra­hydro­carbazolone ester derivatives can also be considered to be synthetic precursors of tetra­cyclic aspidosperma alkaloids, see: Ergün (2007 ▶); For related structures, see: Patır *et al.* (1997 ▶); Hökelek *et al.* (1994 ▶, 1998 ▶, 1999 ▶, 2004 ▶, 2006 ▶); Hökelek & Patır (1999 ▶, 2002 ▶); Çaylak *et al.* (2007 ▶). For bond-length data, see: Allen *et al.* (1987 ▶).
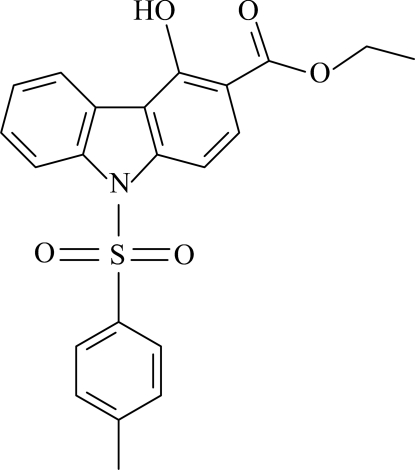


## Experimental

### 

#### Crystal data

C_22_H_19_NO_5_S
*M* *_r_* = 409.44
Monoclinic, 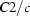

*a* = 23.2155 (12) Å
*b* = 12.3581 (7) Å
*c* = 15.1001 (8) Å
β = 119.656 (1)°
*V* = 3764.7 (4) Å^3^
*Z* = 8
Mo *K*α radiation
μ = 0.21 mm^−1^
*T* = 100 K
0.40 × 0.25 × 0.17 mm

#### Data collection

Bruker Kappa APEXII CCD area-detector diffractometer
Absorption correction: multi-scan (*SADABS*; Bruker, 2005 ▶) *T*_min_ = 0.937, *T*_max_ = 0.962
15402 measured reflections
4658 independent reflections
3276 reflections with *I* > 2σ(*I*)
*R*_int_ = 0.048

#### Refinement

*R*[*F* ^2^ > 2σ(*F* ^2^)] = 0.038
*wR*(*F* ^2^) = 0.092
*S* = 0.94
4658 reflections
292 parameters
H atoms treated by a mixture of independent and constrained refinement
Δρ_max_ = 0.47 e Å^−3^
Δρ_min_ = −0.47 e Å^−3^

### 

Data collection: *APEX2* (Bruker, 2007 ▶); cell refinement: *SAINT* (Bruker, 2007 ▶); data reduction: *SAINT*; program(s) used to solve structure: *SHELXS97* (Sheldrick, 2008 ▶); program(s) used to refine structure: *SHELXL97* (Sheldrick, 2008 ▶); molecular graphics: *ORTEP-3 for Windows* (Farrugia, 1997 ▶); software used to prepare material for publication: *WinGX* (Farrugia, 1999 ▶) and *PLATON* (Spek, 2009 ▶).

## Supplementary Material

Crystal structure: contains datablocks I, global. DOI: 10.1107/S1600536809021035/xu2536sup1.cif

Structure factors: contains datablocks I. DOI: 10.1107/S1600536809021035/xu2536Isup2.hkl

Additional supplementary materials:  crystallographic information; 3D view; checkCIF report

## Figures and Tables

**Table 1:** Hydrogen-bond geometry (Å, °)

*D*—H⋯*A*	*D*—H	H⋯*A*	*D*⋯*A*	*D*—H⋯*A*
O1—H1*A*⋯O4	0.90 (3)	1.73 (2)	2.5746 (18)	156 (2)
C12—H12⋯O1^i^	0.93	2.55	3.410 (2)	154
C16—H16*B*⋯*Cg*4^ii^	0.96	2.91	3.559 (2)	126

Symmetry codes: (i) 
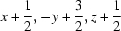
; (ii) 
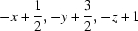
. *Cg*4 is centroid of the C10–C15 ring.

**Fig. 1.**
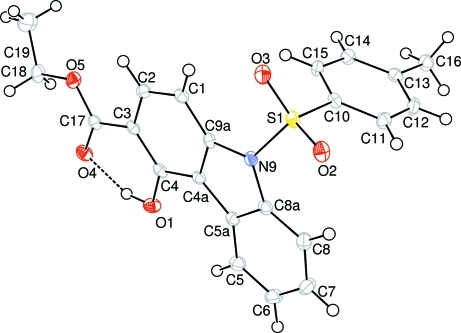
The molecular structure of the title molecule with the atom-numbering scheme. The displacement ellipsoids are drawn at the 50% probability level. Hydrogen bond is shown as dashed line.

**Fig. 2.**
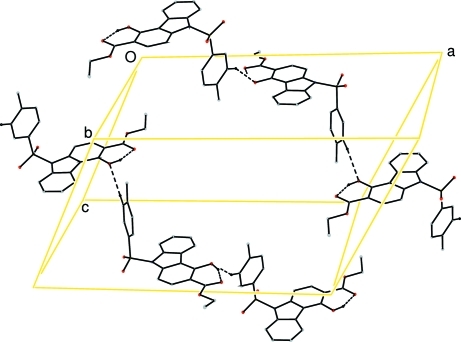
A partial packing diagram for (I). Hydrogen bonds are shown as dashed lines. H atoms not involved in hydrogen bonding have been omitted for clarity.

## supplementary crystallographic information

### Comment

Biologically active compounds, which have tetrahydrocarbazole substructure, have been shown to be useful for the treatment of a variety of medicinal conditions. Tetrahydrocarbazolone derivatives were used in the synthesis of Ondansetron, which is an excellent antiemetic drug inhibiting serotonin 5-HT_3_ receptor (Coates *et al.*, 1987; Gutman & Cyjon, 2006; Molnar *et al.*, 2006). Tetrahydrocarbazolone ester derivatives can also be considered to be synthetic precursors of tetracyclic aspidosperma alkaloids (Ergün, 2007). The structures of tricyclic, tetracyclic and pentacyclic ring systems with dithiolane and other substituents of the tetrahydrocarbazole core, have been the subject of much interest in our laboratory. These include 1,2,3,4-tetrahydrocarbazole-1-spiro-2'-[1,3]dithiolane, (II) (Hökelek *et al.*, 1994), *N*-(2-methoxyethyl)-*N*-{2,3,4,9-tetrahydrospiro[1*H*-carbazole-1, 2-(1,3)dithiolane]-4-yl}benzene-sulfonamide, (III) (Patır *et al.*, 1997), spiro[carbazole-1(2*H*),2'-[1,3]-dithiolan]-4(3*H*)-one, (IV) (Hökelek *et al.*, 1998), 9-acetonyl-3-ethylidene-1,2,3,4-tetrahydrospiro[carbazole-1,2'-[1,3] dithiolan]-4-one, (V) (Hökelek *et al.*, 1999), *N*-(2,2-dimethoxyethyl)-N -{9-methoxymethyl-1,2,3,4-tetrahydrospiro[carbazole-1,2'-[1,3]dithiolan] -4-yl}benzamide, (VI) (Hökelek & Patır, 1999), 3a,4,10,10*b*-tetrahydro-2H -furo[2,3-*a*]carbazol-5(3*H*)-one, (VII) (Çaylak *et al.*, 2007); also the pentacyclic compounds 6-ethyl-4-(2-methoxyethyl)-2,6-methano-5-oxo-hexahydro- pyrrolo(2,3 - d)carbazole-1-spiro-2'-(1,3)dithiolane, (VIII) (Hökelek & Patır, 2002), *N*-(2-benzyloxyethyl)-4,7-dimethyl-6-(1,3-dithiolan-2-yl)-1,2, 3,4,5,6-hexahydro-1,5-methano-2-azocino[4,3-*b*]indol-2-one, (IX) (Hökelek *et al.*, 2004) and 4-ethyl-6,6-ethylenedithio-2-(2-methoxyethyl)-7-methoxy- methylene-2,3,4,5,6,7-hexahydro-1,5-methano-1*H*-azocino[4,3-*b*]indol-3-one, (*X*) (Hökelek *et al.*, 2006). The title compound, (I), may be considered as a synthetic precursor of tetracyclic indole alkaloids of biological interests. The present study was undertaken to ascertain its crystal structure.

The molecule of the title compound (Fig. 1) contains a carbazole skeleton with a tosyl group, where the bond lengths (Allen *et al.*, 1987) and angles are within normal ranges, and generally agree with those in compounds (II)-(*X*). In all structures atom N9 is substituted.

An examination of the deviations from the least-squares planes through individual rings shows that rings A (C1—C4/C4a/C9a), B (C4a/C5a/C8a/N9/C9a), C (C5a/C5—C8/C8a) and D (C10—C15) are planar. The carbazole skeleton, containing the rings A, B and C, is also nearly coplanar [with a maximum deviation of -0.043 (1) Å for atom C4a] with dihedral angles of A/B = 2.32 (6), A/C = 2.94 (5) and B/C = 1.77 (6) °. Ring D is oriented with respect to the planar carbazole skeleton at a dihedral angle of 82.25 (5)°. Intramolecular O—H···O hydrogen bond (Table 1) results in the formation of a planar six-membered ring, E (O1/O4/C3/C4/C17/H1A), which is oriented with respect to the adjacent planar carbazole skeleton at a dihedral angle of 3.06 (4)°. So, they are almost coplanar.

In the crystal structure, intermolecular C—H···O interactions (Table 1) link the molecules into infinite chains (Fig. 2), in which they may be effective in the stabilization of the structure. The π–π contacts between the benzene rings and between the pyrrole and benzene rings, *Cg*1—*Cg*1^i^ and *Cg*2—*Cg*1^i^, [symmetry code:(i) -*x*, 1 - *y*, -*z*, where *Cg*1 and *Cg*2 are centroids of the rings A (C1—C4/C4a/C9a) and B (C4a/C5a/C8a/N9/C9a), respectively] may further stabilize the structure, with centroid-centroid distances of 3.374 (1) and 3.730 (1) Å, respectively. There also exists a weak C—H···π interaction (Table 1).

### Experimental

For the preparation of the title compound, (I), a solution of ethyl-4-oxo-1,2,3,4-tetrahydro-9*H*-carbazole-3-carboxylate (1.25 g, 4.9 mmol) in dichloromethane (25 ml) was cooled to 273 K, and then sodium hydroxide (40%, 5 ml), tetrabutylammonium hydrogen sulfate (0.10 g) and *p*-toluene sulfonyl chloride (0.95 g, 5 mmol) were added. The mixture was stirred for 1 h, and then washed with hydrochloric acid solution (10%, 50 ml), and the organic layer was dried with anhydrous magnesium sulfate. The solvent was evaporated under reduced pressure and the resulting residue was chromatographed using silica gel and ethyl acetate-hexane (1:1). The product was recrystallized from ether (yield; 1.40 g, 71%, m.p. 459 K).

### Refinement

Atoms H1A (for OH), H1, H2, H5, H6, H7 and H8 were located in difference syntheses and refined isotropically. The remaining H atoms were positioned geometrically, with C—H = 0.93, 0.97 and 0.96 Å for aromatic, methylene and methyl H, respectively, and constrained to ride on their parent atoms, with *U*_iso_(H) = *xU*_eq_(C), where *x* = 1.5 for methyl H and *x* = 1.2 for all other H atoms.

### Crystal data

**Table:**

C_22_H_19_NO_5_S	*F*(000) = 1712
*M**_r_* = 409.44	*D*_x_ = 1.445 Mg m^−^^3^
Monoclinic, *C*2/*c*	Mo *K*α radiation, λ = 0.71073 Å
Hall symbol: -C 2yc	Cell parameters from 4061 reflections
*a* = 23.2155 (12) Å	θ = 2.8–28.2°
*b* = 12.3581 (7) Å	µ = 0.21 mm^−^^1^
*c* = 15.1001 (8) Å	*T* = 100 K
β = 119.656 (1)°	Block, colorless
*V* = 3764.7 (4) Å^3^	0.40 × 0.25 × 0.17 mm
*Z* = 8	

### Data collection

**Table:**

Bruker Kappa APEXII CCD area-detector diffractometer	4658 independent reflections
Radiation source: fine-focus sealed tube	3276 reflections with *I* > 2σ(*I*)
graphite	*R*_int_ = 0.048
φ and ω scans	θ_max_ = 28.3°, θ_min_ = 1.9°
Absorption correction: multi-scan (*SADABS*; Bruker, 2005)	*h* = −30→27
*T*_min_ = 0.937, *T*_max_ = 0.962	*k* = −16→16
15402 measured reflections	*l* = −15→20

### Refinement

**Table:**

Refinement on *F*^2^	Primary atom site location: structure-invariant direct methods
Least-squares matrix: full	Secondary atom site location: difference Fourier map
*R*[*F*^2^ > 2σ(*F*^2^)] = 0.038	Hydrogen site location: inferred from neighbouring sites
*wR*(*F*^2^) = 0.092	H atoms treated by a mixture of independent and constrained refinement
*S* = 0.94	*w* = 1/[σ^2^(*F*_o_^2^) + (0.0454*P*)^2^] where *P* = (*F*_o_^2^ + 2*F*_c_^2^)/3
4658 reflections	(Δ/σ)_max_ < 0.001
292 parameters	Δρ_max_ = 0.47 e Å^−^^3^
0 restraints	Δρ_min_ = −0.47 e Å^−^^3^

### Special details

**Table:**

Geometry. All e.s.d.'s (except the e.s.d. in the dihedral angle between two l.s. planes) are estimated using the full covariance matrix. The cell e.s.d.'s are taken into account individually in the estimation of e.s.d.'s in distances, angles and torsion angles; correlations between e.s.d.'s in cell parameters are only used when they are defined by crystal symmetry. An approximate (isotropic) treatment of cell e.s.d.'s is used for estimating e.s.d.'s involving l.s. planes.
Refinement. Refinement of *F*^2^ against ALL reflections. The weighted *R*-factor *wR* and goodness of fit *S* are based on *F*^2^, conventional *R*-factors *R* are based on *F*, with *F* set to zero for negative *F*^2^. The threshold expression of *F*^2^ > σ(*F*^2^) is used only for calculating *R*-factors(gt) *etc*. and is not relevant to the choice of reflections for refinement. *R*-factors based on *F*^2^ are statistically about twice as large as those based on *F*, and *R*- factors based on ALL data will be even larger.

### Fractional atomic coordinates and isotropic or equivalent isotropic displacement parameters (Å^2^)

**Table:**

	*x*	*y*	*z*	*U*_iso_*/*U*_eq_	
S1	0.212099 (19)	0.58317 (3)	0.16682 (3)	0.01509 (11)	
O1	−0.06829 (6)	0.61107 (10)	0.11936 (9)	0.0201 (3)	
H1A	−0.0974 (11)	0.5606 (17)	0.1153 (16)	0.048 (7)*	
O2	0.23596 (5)	0.66022 (9)	0.12235 (8)	0.0199 (3)	
O3	0.21467 (5)	0.47046 (9)	0.14899 (9)	0.0195 (3)	
O4	−0.12557 (5)	0.43127 (10)	0.11530 (9)	0.0208 (3)	
O5	−0.07141 (5)	0.27539 (9)	0.13393 (9)	0.0193 (3)	
C1	0.08618 (8)	0.42675 (13)	0.13014 (12)	0.0144 (3)	
H1	0.1207 (8)	0.3866 (13)	0.1312 (12)	0.018 (4)*	
C2	0.03301 (8)	0.37694 (14)	0.12958 (12)	0.0149 (3)	
H2	0.0310 (8)	0.2986 (14)	0.1300 (13)	0.018 (5)*	
C3	−0.01988 (7)	0.43566 (13)	0.12663 (11)	0.0141 (3)	
C4	−0.01892 (7)	0.54881 (14)	0.12407 (11)	0.0149 (3)	
C4A	0.03495 (7)	0.60124 (13)	0.12560 (11)	0.0138 (3)	
C5	0.01396 (8)	0.80917 (14)	0.11276 (13)	0.0193 (4)	
H5	−0.0270 (9)	0.8081 (15)	0.1138 (14)	0.027 (5)*	
C5A	0.04860 (8)	0.71408 (13)	0.12014 (12)	0.0150 (3)	
C6	0.04061 (9)	0.90650 (14)	0.10672 (13)	0.0230 (4)	
H6	0.0177 (8)	0.9736 (14)	0.1024 (12)	0.016 (4)*	
C7	0.10079 (9)	0.91078 (15)	0.10819 (14)	0.0233 (4)	
H7	0.1175 (9)	0.9779 (15)	0.1057 (14)	0.031 (5)*	
C8	0.13612 (9)	0.81785 (14)	0.11499 (13)	0.0198 (4)	
H8	0.1773 (9)	0.8231 (14)	0.1173 (14)	0.029 (5)*	
C8A	0.10910 (8)	0.71993 (13)	0.12027 (12)	0.0156 (3)	
C9A	0.08633 (7)	0.53939 (13)	0.12851 (11)	0.0135 (3)	
N9	0.13218 (6)	0.61218 (10)	0.12305 (10)	0.0141 (3)	
C10	0.25002 (7)	0.60690 (13)	0.29814 (12)	0.0142 (3)	
C11	0.28921 (8)	0.69829 (13)	0.33915 (13)	0.0183 (4)	
H11	0.2962	0.7460	0.2977	0.022*	
C12	0.31773 (8)	0.71715 (14)	0.44291 (13)	0.0193 (4)	
H12	0.3448	0.7773	0.4711	0.023*	
C13	0.30678 (8)	0.64815 (13)	0.50581 (12)	0.0161 (4)	
C14	0.26657 (8)	0.55758 (13)	0.46226 (13)	0.0175 (4)	
H14	0.2585	0.5109	0.5032	0.021*	
C15	0.23858 (8)	0.53610 (13)	0.35925 (12)	0.0171 (3)	
H15	0.2124	0.4750	0.3312	0.020*	
C16	0.33734 (8)	0.67164 (14)	0.61810 (12)	0.0203 (4)	
H16A	0.3125	0.6354	0.6446	0.030*	
H16B	0.3367	0.7482	0.6282	0.030*	
H16C	0.3823	0.6463	0.6530	0.030*	
C17	−0.07694 (8)	0.38237 (14)	0.12438 (12)	0.0160 (3)	
C18	−0.12612 (8)	0.21883 (14)	0.13567 (14)	0.0220 (4)	
H18A	−0.1649	0.2181	0.0682	0.026*	
H18B	−0.1376	0.2543	0.1821	0.026*	
C19	−0.10271 (10)	0.10619 (15)	0.17057 (17)	0.0345 (5)	
H19A	−0.1368	0.0664	0.1747	0.052*	
H1B	−0.0636	0.1083	0.2365	0.052*	
H19C	−0.0927	0.0715	0.1230	0.052*	

### Atomic displacement parameters (Å^2^)

**Table:**

	*U*^11^	*U*^22^	*U*^33^	*U*^12^	*U*^13^	*U*^23^
S1	0.01240 (19)	0.0197 (2)	0.0141 (2)	−0.00144 (17)	0.00723 (16)	−0.00225 (17)
O1	0.0172 (6)	0.0204 (7)	0.0258 (7)	0.0053 (5)	0.0130 (6)	0.0031 (5)
O2	0.0179 (6)	0.0270 (7)	0.0175 (6)	−0.0049 (5)	0.0107 (5)	−0.0004 (5)
O3	0.0167 (6)	0.0212 (6)	0.0223 (6)	−0.0009 (5)	0.0109 (5)	−0.0066 (5)
O4	0.0151 (6)	0.0263 (7)	0.0215 (6)	0.0020 (5)	0.0094 (5)	0.0029 (5)
O5	0.0166 (6)	0.0196 (6)	0.0237 (6)	−0.0042 (5)	0.0115 (5)	−0.0002 (5)
C1	0.0131 (8)	0.0174 (9)	0.0119 (8)	0.0020 (7)	0.0055 (7)	−0.0004 (7)
C2	0.0173 (8)	0.0151 (9)	0.0110 (8)	0.0009 (7)	0.0060 (7)	0.0003 (7)
C3	0.0127 (7)	0.0194 (9)	0.0094 (7)	0.0011 (7)	0.0048 (6)	0.0008 (6)
C4	0.0128 (8)	0.0211 (9)	0.0099 (8)	0.0026 (7)	0.0048 (6)	0.0012 (6)
C4A	0.0136 (7)	0.0157 (8)	0.0101 (7)	0.0016 (6)	0.0044 (6)	0.0009 (6)
C5	0.0188 (9)	0.0192 (9)	0.0188 (9)	0.0034 (7)	0.0084 (7)	0.0007 (7)
C5A	0.0156 (8)	0.0172 (9)	0.0102 (8)	−0.0004 (7)	0.0048 (7)	−0.0001 (6)
C6	0.0257 (9)	0.0161 (9)	0.0228 (9)	0.0039 (8)	0.0086 (8)	0.0004 (8)
C7	0.0292 (10)	0.0153 (9)	0.0225 (9)	−0.0042 (8)	0.0106 (8)	0.0001 (8)
C8	0.0190 (9)	0.0216 (9)	0.0180 (9)	−0.0033 (8)	0.0084 (7)	−0.0005 (7)
C8A	0.0156 (8)	0.0168 (9)	0.0113 (8)	0.0014 (7)	0.0044 (7)	−0.0009 (7)
C9A	0.0115 (7)	0.0185 (9)	0.0099 (7)	−0.0017 (6)	0.0049 (6)	−0.0017 (6)
N9	0.0115 (6)	0.0153 (7)	0.0148 (7)	−0.0009 (5)	0.0060 (6)	−0.0007 (6)
C10	0.0113 (7)	0.0186 (9)	0.0121 (8)	0.0008 (6)	0.0052 (6)	−0.0013 (6)
C11	0.0182 (8)	0.0190 (9)	0.0181 (8)	−0.0030 (7)	0.0093 (7)	0.0019 (7)
C12	0.0179 (8)	0.0175 (9)	0.0195 (9)	−0.0059 (7)	0.0070 (7)	−0.0024 (7)
C13	0.0146 (8)	0.0181 (9)	0.0160 (8)	0.0020 (7)	0.0079 (7)	0.0006 (7)
C14	0.0164 (8)	0.0195 (9)	0.0182 (9)	−0.0016 (7)	0.0097 (7)	0.0025 (7)
C15	0.0145 (8)	0.0150 (8)	0.0200 (9)	−0.0034 (7)	0.0072 (7)	−0.0023 (7)
C16	0.0208 (9)	0.0221 (10)	0.0159 (9)	−0.0022 (7)	0.0075 (7)	0.0000 (7)
C17	0.0158 (8)	0.0208 (9)	0.0091 (7)	0.0001 (7)	0.0045 (7)	0.0010 (7)
C18	0.0173 (8)	0.0254 (10)	0.0244 (9)	−0.0073 (8)	0.0111 (8)	−0.0009 (8)
C19	0.0290 (10)	0.0273 (11)	0.0485 (13)	−0.0055 (9)	0.0202 (10)	0.0059 (10)

### Geometric parameters (Å, °)

**Table:**

S1—O2	1.4257 (11)	C8—H8	0.942 (18)
S1—O3	1.4255 (12)	C8A—C8	1.383 (2)
S1—N9	1.6715 (13)	C9A—C1	1.392 (2)
S1—C10	1.7508 (16)	N9—C9A	1.4268 (19)
O1—C4	1.3527 (18)	N9—C8A	1.428 (2)
O1—H1A	0.90 (2)	C10—C11	1.389 (2)
O4—C17	1.2269 (18)	C10—C15	1.389 (2)
O5—C17	1.3293 (19)	C11—H11	0.9300
O5—C18	1.4613 (19)	C12—C11	1.386 (2)
C1—C2	1.376 (2)	C12—C13	1.390 (2)
C1—H1	0.937 (16)	C12—H12	0.9300
C2—H2	0.970 (17)	C14—C13	1.396 (2)
C3—C2	1.408 (2)	C14—H14	0.9300
C3—C4	1.399 (2)	C15—C14	1.383 (2)
C3—C17	1.465 (2)	C15—H15	0.9300
C4A—C4	1.398 (2)	C16—C13	1.507 (2)
C4A—C9A	1.399 (2)	C16—H16A	0.9600
C4A—C5A	1.441 (2)	C16—H16B	0.9600
C5—H5	0.959 (17)	C16—H16C	0.9600
C5A—C5	1.397 (2)	C18—C19	1.492 (2)
C5A—C8A	1.405 (2)	C18—H18A	0.9700
C6—C5	1.376 (2)	C18—H18B	0.9700
C6—C7	1.387 (2)	C19—H19A	0.9600
C6—H6	0.970 (17)	C19—H1B	0.9600
C7—H7	0.925 (19)	C19—H19C	0.9600
C8—C7	1.385 (2)		
			
O2—S1—N9	106.52 (7)	C4A—C9A—N9	107.62 (14)
O2—S1—C10	109.07 (7)	C8A—N9—S1	122.58 (10)
O3—S1—O2	120.09 (7)	C9A—N9—S1	123.76 (11)
O3—S1—N9	106.08 (7)	C9A—N9—C8A	108.04 (12)
O3—S1—C10	109.45 (7)	C11—C10—S1	119.51 (12)
N9—S1—C10	104.46 (7)	C11—C10—C15	120.95 (15)
C4—O1—H1A	101.4 (14)	C15—C10—S1	119.51 (12)
C17—O5—C18	116.08 (13)	C10—C11—H11	120.6
C2—C1—C9A	117.22 (15)	C12—C11—C10	118.81 (15)
C2—C1—H1	121.4 (10)	C12—C11—H11	120.6
C9A—C1—H1	121.4 (10)	C11—C12—C13	121.51 (15)
C1—C2—C3	122.37 (16)	C11—C12—H12	119.2
C1—C2—H2	119.6 (10)	C13—C12—H12	119.2
C3—C2—H2	118.1 (10)	C12—C13—C14	118.40 (15)
C2—C3—C17	122.25 (15)	C12—C13—C16	120.46 (15)
C4—C3—C2	119.37 (15)	C14—C13—C16	121.14 (14)
C4—C3—C17	118.37 (14)	C13—C14—H14	119.5
O1—C4—C3	123.04 (14)	C15—C14—C13	121.08 (15)
O1—C4—C4A	117.70 (15)	C15—C14—H14	119.5
C4A—C4—C3	119.26 (14)	C10—C15—H15	120.4
C4—C4A—C5A	131.87 (15)	C14—C15—C10	119.24 (15)
C4—C4A—C9A	119.28 (15)	C14—C15—H15	120.4
C9A—C4A—C5A	108.78 (14)	C13—C16—H16A	109.5
C5A—C5—H5	121.5 (11)	C13—C16—H16B	109.5
C6—C5—C5A	118.72 (16)	C13—C16—H16C	109.5
C6—C5—H5	119.7 (11)	H16A—C16—H16B	109.5
C5—C5A—C4A	133.16 (15)	H16A—C16—H16C	109.5
C5—C5A—C8A	119.49 (15)	H16B—C16—H16C	109.5
C8A—C5A—C4A	107.32 (14)	O4—C17—O5	122.42 (15)
C5—C6—C7	120.98 (17)	O4—C17—C3	123.56 (16)
C5—C6—H6	120.1 (9)	O5—C17—C3	114.02 (14)
C7—C6—H6	118.9 (9)	O5—C18—C19	106.54 (14)
C6—C7—H7	118.2 (12)	O5—C18—H18A	110.4
C8—C7—C6	121.62 (17)	O5—C18—H18B	110.4
C8—C7—H7	120.2 (12)	C19—C18—H18A	110.4
C7—C8—H8	119.9 (11)	C19—C18—H18B	110.4
C8A—C8—C7	117.43 (16)	H18A—C18—H18B	108.6
C8A—C8—H8	122.6 (11)	C18—C19—H19A	109.5
C5A—C8A—N9	108.19 (13)	C18—C19—H1B	109.5
C8—C8A—C5A	121.76 (15)	C18—C19—H19C	109.5
C8—C8A—N9	130.01 (15)	H19A—C19—H1B	109.5
C1—C9A—N9	129.78 (14)	H19A—C19—H19C	109.5
C1—C9A—C4A	122.49 (14)	H1B—C19—H19C	109.5
			
O2—S1—N9—C8A	−45.91 (13)	C5A—C4A—C9A—C1	−177.57 (14)
O2—S1—N9—C9A	163.72 (12)	C4—C4A—C9A—N9	176.41 (13)
O3—S1—N9—C8A	−174.93 (11)	C5A—C4A—C9A—N9	−1.04 (17)
O3—S1—N9—C9A	34.70 (14)	C4A—C5A—C5—C6	−178.59 (16)
C10—S1—N9—C8A	69.46 (13)	C8A—C5A—C5—C6	−0.6 (2)
C10—S1—N9—C9A	−80.91 (13)	C5—C5A—C8A—C8	1.1 (2)
O2—S1—C10—C11	7.52 (15)	C4A—C5A—C8A—C8	179.59 (14)
O2—S1—C10—C15	−174.74 (12)	C5—C5A—C8A—N9	−176.99 (14)
O3—S1—C10—C11	140.73 (13)	C4A—C5A—C8A—N9	1.49 (17)
O3—S1—C10—C15	−41.54 (14)	C7—C6—C5—C5A	−0.2 (3)
N9—S1—C10—C11	−106.05 (13)	C5—C6—C7—C8	0.5 (3)
N9—S1—C10—C15	71.69 (14)	C8A—C8—C7—C6	0.0 (3)
C18—O5—C17—O4	−1.5 (2)	N9—C8A—C8—C7	176.83 (15)
C18—O5—C17—C3	177.97 (13)	C5A—C8A—C8—C7	−0.8 (2)
C17—O5—C18—C19	−167.19 (14)	N9—C9A—C1—C2	−176.19 (15)
C9A—C1—C2—C3	0.5 (2)	C4A—C9A—C1—C2	−0.5 (2)
C4—C3—C2—C1	0.1 (2)	S1—N9—C8A—C5A	−156.54 (11)
C17—C3—C2—C1	179.00 (15)	S1—N9—C8A—C8	25.6 (2)
C2—C3—C4—O1	178.80 (14)	C9A—N9—C8A—C5A	−2.15 (16)
C2—C3—C4—C4A	−0.7 (2)	C9A—N9—C8A—C8	179.97 (16)
C17—C3—C4—O1	−0.2 (2)	S1—N9—C9A—C1	−27.8 (2)
C17—C3—C4—C4A	−179.66 (14)	S1—N9—C9A—C4A	155.97 (11)
C2—C3—C17—O4	−175.21 (15)	C8A—N9—C9A—C1	178.15 (15)
C2—C3—C17—O5	5.3 (2)	C8A—N9—C9A—C4A	1.96 (16)
C4—C3—C17—O4	3.7 (2)	S1—C10—C15—C14	−177.47 (12)
C4—C3—C17—O5	−175.75 (13)	C11—C10—C15—C14	0.2 (2)
C9A—C4A—C4—O1	−178.80 (14)	S1—C10—C11—C12	178.63 (12)
C5A—C4A—C4—O1	−2.0 (3)	C15—C10—C11—C12	0.9 (2)
C9A—C4A—C4—C3	0.7 (2)	C13—C12—C11—C10	−1.4 (3)
C5A—C4A—C4—C3	177.46 (16)	C11—C12—C13—C14	0.7 (2)
C4—C4A—C5A—C5	0.9 (3)	C11—C12—C13—C16	−178.83 (15)
C4—C4A—C5A—C8A	−177.30 (16)	C15—C14—C13—C12	0.5 (2)
C9A—C4A—C5A—C5	177.91 (17)	C15—C14—C13—C16	−179.98 (15)
C9A—C4A—C5A—C8A	−0.28 (17)	C10—C15—C14—C13	−1.0 (2)
C4—C4A—C9A—C1	−0.1 (2)		

### Hydrogen-bond geometry (Å, °)

**Table:**

*D*—H···*A*	*D*—H	H···*A*	*D*···*A*	*D*—H···*A*
O1—H1A···O4	0.90 (3)	1.73 (2)	2.5746 (18)	156 (2)
C12—H12···O1^i^	0.93	2.55	3.410 (2)	154
C16—H16B···Cg4^ii^	0.96	2.91	3.559 (2)	126

Symmetry codes: (i) *x*+1/2, −*y*+3/2, *z*+1/2; (ii) −*x*+1/2, −*y*+3/2, −*z*+1.
